# Designing New Sport Supplements Based on *Aronia melanocarpa* and Bee Pollen to Enhance Antioxidant Capacity and Nutritional Value

**DOI:** 10.3390/molecules28196944

**Published:** 2023-10-05

**Authors:** Adrian Tirla, Adrian Vasile Timar, Anca Becze, Adriana Ramona Memete, Simona Ioana Vicas, Mihaela Simona Popoviciu, Simona Cavalu

**Affiliations:** 1 Doctoral School of Biomedical Sciences, Faculty of Medicine and Pharmacy, University of Oradea, P-ta 1 Decembrie 10, 410087 Oradea, Romania; adriantirla18@gmail.com; 2 Faculty of Environmental Protection, University of Oradea, 26 Gen. Magheru Street, 410048 Oradea, Romania; atimar@uoradea.ro (A.V.T.); adrianamemete@yahoo.com (A.R.M.); 3 INCDO-INOE 2000 Subsidiary Research Institute for Analytical Instrumentation ICIA, 67 Donath Street, 400293 Cluj-Napoca, Romania; anca.naghiu@icia.ro; 4 Faculty of Medicine and Pharmacy, University of Oradea, P-ta 1 Decembrie 10, 410087 Oradea, Romania; elapopoviciu@yahoo.com

**Keywords:** natural supplement, sport nutrition, bioactive compounds, antioxidants, *Aronia melanocarpa*, bee pollen

## Abstract

With a high number of athletes using sport supplements targeting different results, the need for complex, natural and effective formulations represents an actual reality, while nutrition dosing regimens aiming to sustain the health and performance of athletes are always challenging. In this context, the main goal of this study was to elaborate a novel and complex nutraceutical supplement based on multiple bioactive compounds extracted from *Aronia melanocarpa* and bee pollen, aiming to support physiological adaptations and to minimize the stress generated by intense physical activity in the case of professional or amateur athletes. Our proposed formulations are based on different combinations of Aronia and bee pollen (A1:P1, A1:P2 and A2:P1), offering personalized supplements designed to fulfill the individual requirements of different categories of athletes. The approximate composition, fatty acid profile, identification and quantification of individual polyphenols, along with the antioxidant capacity of raw biological materials and different formulations, was performed using spectrophotometric methods, GS-MS and HPLC-DAD-MS-ESI+. In terms of antioxidant capacity, our formulations based on different ratios of bee pollen and Aronia were able to act as complex and powerful antioxidant products, highlighted by the synergic or additional effect of the combinations. Overall, the most powerful synergism was obtained for the A1:P2 formulation.

## 1. Introduction

Sport nutrition is an essential link in sustaining the health and performance of athletes. Considering this demand, there is high interest in developing tailored supplements for the express needs of athletes. In the past, the academic community and the Olympic committee elaborated guidelines and recommendations regarding nutritional needs and the effects of various nutrients and substances [1]. According to these recommendations, manufacturers released multiple industrial products into the market, usually aiming towards a solitary objective concerning athletes’ metabolism. Nowadays, the perspective has changed slightly, with the promotion of a more holistic approach which includes the use of natural or naturally derived products with the supposed advantages of higher bioavailability and adjuvant effects of specific cofactors [2,3]. This can explain the mixed results of several studies using different sources of the same nutrient [4]. With a high percentage of athletes using sport supplements targeting different effects, the need for complex and effective formulations is an actual reality.

Bee pollen is constituted of floral pollen mixed with flower nectar and enzymes secreted by the salivary glands of honeybees, *Apis melifera*. It has a complex chemical composition consisting of proteins, amino acids, carbohydrates and fatty acids, along with micronutrients such as minerals and vitamins [5]. It has been part of the human diet for thousands of years, representing 22% of the Tyrolean iceman’s colon content, who is supposed to have lived 5300 years ago [6]. However, nowadays, allergic reactions might represent a challenge in pollen consumption [7]. There are multiple proven positive effects of bee pollen, such as antioxidant, anti-inflammatory, antiaging, antilipidemic, anticancer, antiallergic and antimicrobial activities, with benefits in multiple pathologies such as prostate hyperplasia, breast cancer, allergies, diabetes, immune function and skin-related problems [8,9]. The potential of bee pollen in sport nutrition is considerable due to the balanced ratio of macronutrients and the potent effects of its micronutrients; of all the bee products, it is the most abundant in proteins and polyphenols [8]. This makes it a perfect candidate for sport nutrition, with an optimum ratio of macronutrients; it is made up of about 20% proteins and it has an Amino Acid Score (AAS) up to 100%, an EAAI between 63 and 78% [10,11], and up to 55% carbohydrates [12].

There are a few studies assessing the effects of bee pollen on athletes [13,14,15]. A significant reduction in upper respiratory infections was found in a pollen supplementation group of adolescent swimmers [13]. Animal model studies such as mustard bee pollen supplementation in rats have yielded promising results, such as myostatin reduction and increased antioxidant capacity and mitochondrial function [16]. Increased muscle anabolism was the outcome of pollen supplementation in old, malnourished rats via MTOR activation with a linear dose–effect curve [17]. Bee pollen exerted a protective effect on induced acute kidney injuries in rats [18], and it might have a positive effect on exercise-induced kidney injuries. Improved physical fitness and nutrient uptake was seen in Arabian horses after 42 days of pollen-based product supplementation [19].

Aronia (*Aronia melanocarpa)* is known for its impressive phenolic content and multiple clinical benefits, such as antioxidant, anti-inflammatory, hypotensive, antiviral, anticancer, antiplatelet, antidiabetic and antiatherosclerotic activities [20,21]. Intense sport activity is demonstrated to generate significant oxidative stress, and NADPH oxidase NOX2 is the most active generator of reactive oxygen species (ROS) during intense muscular activity [22]. The response of the body to maintain an optimum redox balance is to develop an intrinsic antioxidant response capacity composed mainly of enzymatic antioxidants, such as SOD, CAT, GPX, TRX, and PRX, and nonenzymatic antioxidants, such as GSH, uric acid, bilirubin, vitamin E, vitamin C, and others [22]. Exercise beyond the neutralizing potential of intrinsic antioxidants can lead to the accumulation of ROS, resulting in muscular fatigue and poor muscle performance. Extrinsic antioxidant supplementation might improve peak sports performance by enhancing recovery and preventing tissue damage, especially in elite athletes [23].

Stankiewicz et al. [24], demonstrated in a double-blind randomized trial the positive effects of lyophilized *Aronia melanocarpa* supplementation on antioxidant and anti-inflammatory markers in football players after maximal aerobic exercise. Another study published in 2021 by Cikiriz concluded improvements in the redox state, hematological profile, lipid profile and body composition of handball players after 12 weeks of Aronia supplementation [25], while the antioxidant effects of Aronia juice on rowers was evaluated in a study published by Pilaczynska-Szczesniak et al., 2005 [26], showing positive results. Overall, Aronia might be an important ingredient in any athlete’s diet, considering the plethora of bioactive ingredients. Athletes from several different disciplines (rowers, footballers, handballers, triathletes and runners) can benefit from polyphenol-rich supplementation to regulate various mechanisms associated with exercise performance, as antioxidants have also been reported to blunt training adaptations and accelerate recovery after strenuous exercise [23]. Unlike the general population, athletes might be more dependent on functional foods and supplements. They need a meal packed with high amounts of nutritional elements such as vitamins, minerals, essential amino acids, fatty acids and antioxidants. This meal should have a balanced ratio of carbohydrates–CHO proteins and lipids, while still being low in calories. Fast recovery is essential in competitions, especially after weight loss programs and between bouts when limited time is available to prepare for the next game.

Therefore, the need for diet personalization is compulsory considering the body mass and composition of each individual and the nature of the sport practiced.

The aim of this study is to combine multiple bioactive compounds (proteins, carbohydrates, vitamins, minerals and antioxidants) extracted from *Aronia melanocarpa* and bee pollen in a complex nutraceutical supplement intended to support the physiological adaptations and to minimize the stress generated by intense physical activity in the case of professional and amateur athletes [27]. The proposed formulations are based on different ratios of *Aronia melanocarpa* dried fruits and bee pollen extracts (A2:P1, A1:P1 and A1:P2), aiming to offer a personalized supplement designed to fulfill the individual requirements of different categories of athletes. Different ratios were selected to find which combination can offer the best synergism in terms of antioxidant capacity, along with necessary protein intake; it is well known that oxidative stress and inflammation are important factors closely related to muscle catabolism.

## 2. Results

### 2.1. Characterization of Raw Biological Material

#### Proximate Composition: Total Carbohydrate, Lipid, Protein, Mineral Content and Fatty Acids Profile in Aronia and Bee Pollen Samples

Table 1 shows the proximate composition of Dried Aronia fruits, bee pollen and different combinations proposed for the development of the supplements, along with the calculated energy value. The energy content of a mixture was calculated based on the general factors of fats, proteins and carbohydrates.

The mineral content and fatty acids profile of the raw biological materials as well as the combinations are presented in Table 2 and Table 3, respectively.

The unsaturated index, UI, represents the sum of each PUFA multiplied by its number of pentadienyl groups [28]. The atherogenic potential of fatty acids can be evaluated by the index of atherogenicity, which represents the ratio of the weighted sum of main saturated fats and the sum of unsaturated fats, as illustrated by the formula IA = (C12:0 + C16:0 + 4 × C14:0) ÷ [ΣMUFA + Σ(n − 6) + Σ(n − 3)] [29].

The index of thrombogenicity represents the ratio between prothrombogenic and antithrombogenic fatty acids and is calculated with this formula: TI = (C14:0 + C16:0 + C18:0) ÷ [0.5 × ΣMUFA + 0.5 × Σ(n − 6) + 3 × Σ(n − 3) + Σ(n − 3) ÷ Σ(n − 6)].

The hypocholesterolemic/hypercholesterolemic (HH) ratio is an optimized version of PUFA/SFA and is defined by this formula: h/H = (C18:1 + PUFA) ÷ (C12:0 + C14:0 + C16:0).

### 2.2. Structural Characterization via FTIR Spectroscopy

**Figure 1:**
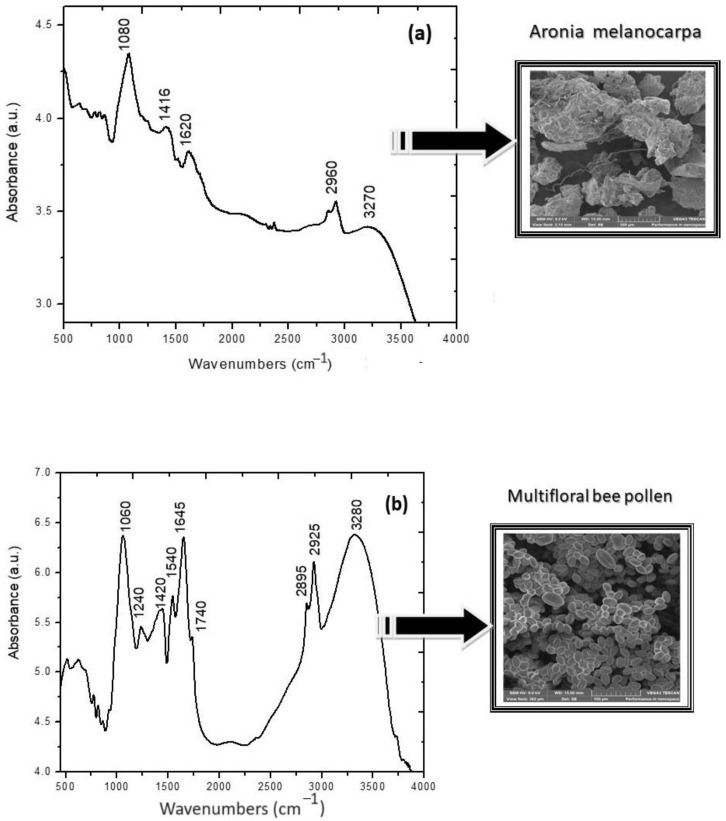
FTIR spectra of Dried Aronia fruits (**a**) and bee pollen (**b**) were recorded using an ATR accessory, along with the ultrastructural details captured via SEM.

The vibrational features of Aronia and pollen samples are presented in Figure 1a,b, along with the microscopic details.

The most prominent vibrational features and characteristic frequencies of functional groups in *A. melanocarpa* dried fruits are Amide I at 1620 cm^−1^ originating from C=O stretching vibrations, amide II at 1416 cm^−1^ from the N-H bending coupled with C-N stretching vibrations, and the strong band characteristic for polysaccharides at 1080 cm^−1^, which are, respectively, the -C-O bonds of cyclic ether and alcoholic groups from polyphenols. In the high wavenumber region, the appearance of a shoulder at 3280 cm^−1^ is due to the stretching and flexion of OH groups and weak and sharp peaks at 2895/2925 cm^−1^ possibly originating from CH_2_ groups (C-H stretching vibrations). These values are similar to previously reported data and vibrational features of Chokeberry fruits that originated in Serbia [30]. The main fingerprints of bee pollen noticed at around 1060 cm^−1^ are attributed to several overlapping signals of sugars, due to stretching -C-O as well as N-H and C-H deformation vibrations with proteins, and a strong amide I band at 1645 cm^−1^ characterized by the overlapping effects of several vibrations related to proteins and lipids, primarily due to the carbonyl group C=O stretching vibrations of the ester bond, which is directly related to the protein backbone conformation. Amide II band is visible as a small peak at 1540 cm^−1^ due to protein-related N-H bending and C-N stretching vibrations, which are overlapped by the lipid-based C=C stretching of aromatic structures, while amide III at 1240 cm^−1^ represents mainly the combination between N-H deformation and C-N stretching vibrations. In the high wavenumber region, the bands observed at 2925 and 2895 cm^−1^ correspond to the asymmetric and symmetric stretching vibrations of CH_3_, which are non-specific vibrational signals, while the broad absorption observed around 3280 cm^−1^ is assigned to both N-H stretching vibrations of the protein structure (amide A band). The results are in concordance with previously reported ones [31,32], confirming the high content of proteins and carbohydrates.

### 2.3. Determination of Bio-Active Compounds in Aronia and Bee Pollen Samples

The total content of bioactive compounds in raw materials is presented in Table 4, as determined using spectrophotometric methods.

### 2.4. Identification and Quantification of Individual Polyphenols via HPLC-DAD-MS-ESI+

In the Aronia sample, 13 phenolic compounds were identified (Table 5), belonging to phenolic acids and flavonoids classes. Three hydroxybenzoic acids (compounds **1**–**3**) and four hydroxycinnamic acids (compounds **7**–**10**) were found among the phenolic acids. Three anthocyanins were isolated and identified from the flavonoid class, which are represented by glycosylated forms of cyanidin, namely Cyanidin-glucoside (compound **4**), Cyanidin-arabinoside (compound **5**) and Cyanidin-xyloside (compound **5**). The separated and identified flavonols are represented by the glycosylated quercetin derivatives, rutin (compound **11**), Quercetin glucosides (compound **12**) and the quercetin aglycone (compound **13**). Quantitative data on the phenolic compounds expressed as µg/g dw from the Aronia extract and the elution order are presented in Table 4, as well as HPLC chromatograms of the Aronia sample recorded at 280 nm, 340 nm and 520 nm (Figure S1, Supplementary Material).

In dried Aronia fruits, hydroxinamic acids were predominant (73.42% of total phenols), from which 5-Feruloylquinic acid (compound **10**) was present in the highest amount (9.049 ± 0.08 mg/g dw), followed by caffeic acid and chlorogenic acid (4.971 ± 0.2 mg/g dw and 3.421 ± 0.2 mg/g dw, respectively). Compounds **9** and **10** presented a maximum UV spectrum at 333 nm, while the MS spectra exhibited [M+H]^+^ at *m*/*z* 369, indicating the presence of Feruloylquinic acid isomers. Quinic acid can form esters with ferulic acid, generating various isomers known as Feruloylquinic acid. Similar results were obtained for the Feruloylquinic acid isomers in mulberry fruits [33].

From the flavonols class, the aglycon quercetin (compound **13**) and its conjugated forms linked to sugars were detected. The UV-Vis spectrum of quercetin presented maximum absorption values at 356 and 251 nm at *m/z* 303. The other two quercetin derivatives (compounds **11** and **12**) show one ionic fragment at *m*/*z* 303, which is specific to quercetin aglycon and other fragments corresponding to the sugar moiety. In the Aronia sample, we noticed a higher level of Quercetin glucosides compared to those of rutin and aglycon (Table 5).

Pollen is also a significant source of phenolic compounds, with 16 flavonoids and phenolic acids being identified and quantified (Figure S2, Supplementary Material). Flavonols exhibited the highest concentrations among all the bioactive compounds in Pollen, representing 47.88% of the total phenols. However, phenolic acids, both hydroxybenzoic acids and hydroxycinnamic acids, account for only 6.40% and 1.66 of the total phenolic compounds in bee pollen. The flavonol derivatives such as quercetin, isorhamnetin and kaempferol glycosides were the main compounds identified in bee pollen. Aylanc et al., 2022, also noticed the presence of these flavonoids, as well as herbacetin glycosides, in bee pollen from northeast Portugal [34].

An important group of compounds found in high concentrations in our bee pollen sample was spermidine (44.058%), which is a polyamine conjugated with hydroxycinnamic acid. The most common hydroxycinnamic acids that are linked with phenylamides are caffeic, ferulic and *p*-coumaric acids [35]. Diferuloyl-coumaroyl spermidine (compound **14**, *m*/*z* 644) was also detected at a very high level in our bee pollen sample (11.926 ± 0.99 mg/g dw). The qualitative and quantitative results (HPLC-DAD-MS (ESI+) regarding the phenolic compounds in our pollen sample are presented in Table 6.

### 2.5. Determination of Antioxidant Capacity of Biological Samples

Four different methods were applied in order to evaluate the antioxidant capacity of the raw biological materials as well as the mixtures containing different ratios of Aronia and pollen (A1:P1; A1:P2; A2:P1). The synergic, antagonist or additional effects were evaluated according to the calculation detailed in Section 4.6. The results and data interpretation are presented in Table 7.

## 3. Discussion

It is generally accepted that the bioavailability of natural supplements is superior to that of the synthetic ones, with a direct impact on athletic performance by worsening various rate-limiting processes, or via an indirect impact by influencing factors such as inflammatory modulation, oxidative stress and signaling pathways for adaptation and ability to support repetitive efforts [23]. The novelty of our study is represented by the unique combination between *Aronia melanocarpa* and bee pollen, providing elements for a personalized sport diet tailored to a particular individual’s requirements. This individualized diet can be adapted to an individual’s needs in various ways, including combining multiple nutrients into a single delivery system or integrating multiple delivery systems containing different nutrients [27]. The individualization of meals based on biometric data has become a significant trend in modern nutrition, advising athletes to consume more or fewer certain nutrients [27].

Proteins are the most common sport supplements used around the world and among the most scientifically supported ones for their anabolic and growth effects [36,37]. In our formulations, the main protein source is constituted of bee pollen with 23.6% protein content. The efficacity of bee pollen protein in muscle anabolism has previously been studied, showing positive results in animal and human studies. The conclusions of a meta-analysis published in 2023 indicated that bee pollen is a potent growth booster for rabbits, also generating a higher plasma antioxidant capacity [38]. Bee pollen is also suggested to be used in the broiler chicken industry with positive results in terms of an immune stimulant, growth and reproductive promoter and intestine health supporter, which makes it similar to antibiotics [39,40,41]. These anabolic effects might be explained by the capacity of bee pollen protein to activate the mTOR pathway [17]. Taking into consideration the results of our study, we can state that in terms of protein content, the most favorable formulation is A1:P2, which is recommended in sports where muscle mass synthesis is required, such as weight lifting or combat sports.

Carbohydrates (CHO) represent the preferential source of energy in the human body and, as a consequence, are widely recommended as effort sustainers [42,43,44]. In sport nutrition, the absorption speed of CHO represents an important feature, and hence, the aim is to bring as much glucose content as possible to the muscle as fast as possible. As a consequence, simple sugars are mostly used as a source of CHO, which is detrimental to polymerized CHO (such as starch). An important limiting factor in the absorption of glucose is the number of SGLT1 receptors present in the intestine wall. In order to bypass these receptors, a combination of SGLT1-dependent (such as glucose or galactose) and SGLT1-independent (such as fructose) ones can be utilized to maximize the absorption rate [45,46]. According to current research, more than 90% of CHO contained in Aronia–pollen mixtures are represented by fructose and glucose [20,47]. In our study, the most amount of CHO was found in bee pollen (31.69%), making the A1:P2 formulation suitable also for endurance sports, such as cycling, rowing or long-distance running.

Fat is an essential element in any diet by providing a source of essential fatty acids and promoting the bioavailability of some fat-soluble nutrients [48,49]. It can also represent a major energy source that can support long endurance, light- and medium-intensity training [50], and fat oxidation, leading to a higher oxygen consumption due to the lower respiratory quotient of 0.7 for fat compared to 1.0 for CHO [51,52]. The general recommendations are that lipids should cover about 20–25% of total caloric intake and most of them should be healthy fats such as MUFA and PUFA to reduce to the minimum saturated fat intake. Special interest is paid to the beneficial effects of Omega-3 fatty acids with positive effects on performance, injury prevention and recovery [1,48]. The WHO recommends an intake of 200–500 mg of Omega-3 fatty acids per day for the general population. Although there are no exact recommendations for athletes, many studies promote the beneficial effect of healthy fats as being cyclooxygenase inhibitors on limiting the inflammation generated via sport activities. This can be translated into a better capacity of the organism to cope with intense repeated training sessions [53]. To evaluate the ratio of healthy and unhealthy fats, several indices are proposed. The fatty acids profiles the of Aronia and pollen samples (displayed in Table 2) can be compared with the general recommendations and the results of other natural products found in the literature. The Omega-6/Omega-3 ratio is one of the simplest ways to evaluate the quality of a fatty product due to the pro-inflammatory effect of Omega-6. High Omega-6 concentrations are found in Western diets, with a ratio of Omega-6/Omega-3 of 16.7:1, and are to be blamed for the negative effects of these diets. The general recommendations are to avoid a ratio above 5:1 or even 2.5:1, with multiple studies supporting the positive effects of these regimens [54]. Our results are within the recommendations ranging between 1.21 and 2.54. In terms of the unsaturation index, the results for our formulations range from 145 for the A2:P1 formulation to 237 for the A1:P2 formulation. Compared to the other results presented in the literature, the values are higher than those found in pork meat or dairy products and comparable with those of other vegetal products such as sea grass, with values ranging from 45 to 368 [55]. Javardi et al., demonstrated the direct correlation between dietary intake, the index of atherogenicity (AI) and the AI values of plasma components as well as the increased body mass index and alteration of plasma lipid profiles [56]. Our results have an IA range between 0.21 and 0.28 for the A2:P1 and A1:P2 formulations, respectively. These findings highlight that all three formulations possess a lower atherogenic value compared to that of dairy or meat products often used in athlete nutrition and a comparable atherogenic value to that of vegetal products such as guar seeds, cumin or seaweed [55].

The relationship between intense exercise and thrombosis is intensely discussed in the literature and leads to mixed conclusions. On one side, exercise improves blood circulation and prevents vascular stasis, one of the three factors of Virchow’s triad, but on the other hand, intense exercise can cause polycythemia, altering the blood coagulability and leading to an increased risk of thrombosis [57]. Another fact to be taken into consideration is that some athletes use banned substances such as anabolic androgenic steroids that promote thrombogenesis, often leading to infarction [58]. The AI results for our formulations are around 0.20, which is better than those of most dairy products (up to 5.04) and comparable with those of crops, such as *Lupinus albus* (0.13–0.18) and *Scabiosa stelata* (0.23), or with shrimp and fish (0.14–0.87) [55]. In terms of the hypocholesterolemic/hypercholesterolemic ratio, our results range from 3.76 to 4.78, showing a hypocholesterolemic potential, exceeding the value of the most studied products [55]. Taking into consideration the high caloric density of lipids, the A2:P1 formula should considered in all sports where weight is extremely important, such as gymnastics, some categories of combat sports or high jumping.

Minerals are essential components of any athlete’s diet, and the insufficiency of any can lead to performance depreciation [59,60]. As shown in Table 2, the studied formulations are rich in potassium, calcium and magnesium, which are required in sport nutrition during intense training, according to recent studies [27], and considered to have a blood-pressure-lowering potential [61]. Although it is well balanced in terms of ratio, the mineral content is not the main feature of our formulations, with it being about 1 kg/day to fulfill the WHO (World Health Organization) recommendations regarding minerals. In our formulations, the Aronia sample proved to be a better source of minerals compared to pollen, and hence, in the cases of high mineral loss situations (for example, intense training, especially on hot days), the A2:P1 formula is recommended.

Oxidative stress occurs when there is an imbalance between free radical production and the ability of the organism to neutralize this [62]. A high level of free radicals causes many biological changes in organisms, which is a major contributing factor in a broad spectrum of diseases [63]. Some free radicals are produced naturally in the body, while others are derived from various sources [64]. During exercise, for example, the metabolism accelerates, oxygen intake rises, and the rate of reactive oxygen species (ROS) formation increases [22,65]. On the one hand, exercise-induced ROS have a detrimental impact, altering the cellular structure and function and, as a result, causing muscle injury, immunological dysfunction and tiredness [22,66]. However, ROS generation following exercise can have favorable effects, such as increasing glycogen resynthesis and decreasing the person’s susceptibility to infection, while initiating and promoting adaptative responses to training, and hence, improving their athletic performance [27,67,68].

Increasing the amounts of exogenous antioxidants in the body through the diet or food supplements is beneficial in terms of antioxidative maintenance. Antioxidants possess the ability to counteract the effects of free radical oxidative damage by preventing or limiting the injury caused by reactive oxygen and reactive nitrogen species [33].

The in vitro methods for the determination of the antioxidant capacity of food components were divided into three groups, depending on the mechanism of action: (i) an assay based on the single electron transfer (SET); (ii) an assay based on hydrogen atom transfer (HAT) and (iii) an assay based on the ability of samples to scavenge reactive oxygen species or reactive nitrogen species (superoxide anion radical, hydroxyl radical, singlet oxygen, peroxynitrite and hydrogen peroxide), which interact with major macromolecules (proteins, lipids and DNA). The methods based on an SET reaction include the following: the Folin–Ciocalteu assay and the TEAC, FRAP, CUPRAC, DPPH, ABTS^•+^ and total antioxidant potential assays using Cu^2+^ as an oxidant. The HAT-based methods include: ORAC, TRAP, the inhibition of induced LDL oxidation, β-Carotene bleaching assays and chemiluminescent assays [69]. The SET mechanism-based approaches are the most frequently employed for assessing the antioxidant capacity of foods.

DPPH is now one of the most widely utilized free-radical-scavenging antioxidant assay. This assay can test a large number of samples and bioactive compounds (polyphenols and flavonoids) fast and accurately. CUPRAC has several more advantages compared to those of DPPH, including the fact that the reagents are more widely available, less expensive and more stable. It is straightforward to use, and there have been no reports of chemical interference in the solutions. In fact, numerous polyphenolics, including flavonoids and phenolic acids, possess a strong correlation with the CUPRAC method. In the FRAP assay performed to retain iron solubility, the transformation from a colorless solution to a blue color occurs under acidic conditions with a pH of 3.6. The FRAP assay offers speedy and consistent results, but there is one fundamental limitation: the antioxidant must be water-soluble. The TEAC or ABTS assay investigates the antioxidant process in dietary components over a wide pH range using lipophilic and hydrophilic compounds in water and organic solvents [70].

In terms of antioxidant capacity, we found different results regarding the individual ingredients, with the pollen scoring better in the DPPH and TEAC assays, while Aronia scored better in the FRAP and CUPRAC assays than the pollen sample did. This might be explained by the different antioxidant compounds, with pollen being very rich in polyphenols, while Aronia is rich in anthocyanins. However, from a practical point of view, in terms of exercise redox biochemistry, there are some limitations due to the chemical heterogeneity of redox biology. Intense sport activity is demonstrated to generate significant oxidative stress, with NADPH oxidase NOX2 being the most active generator of reactive oxygen species (ROS) during intense muscle activity. According to the literature [65], methodological and technical issues might complicate the approach adapted to investigate how exercise alters redox homeostasis.

Regardless of the employed method, we can state that pollen and Aronia complement each other, creating together a complex and powerful antioxidant combination. This affirmation is sustained by the synergistic or additional antioxidant effect of the combinations. Overall, the most powerful synergism was obtained for the A1:P2 formulation, followed by A1:P1.

## 4. Materials and Methods

### 4.1. Biological Material and Experimental Design

**Figure 2:**
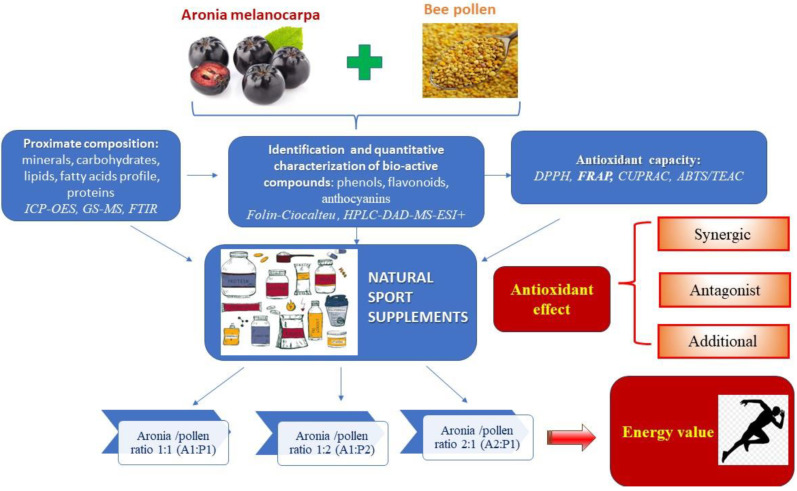
Experimental design: development of novel sport supplements based on *Aronia melanocarpa* (A) and bee pollen (P) at different ratios (A1:P1; A1:P2 and A2:P1, *v*/*v*).

Dried Aronia berries (*Aronia melanocarpa*) and multifloral bee pollen were purchased in July 2022 from a local market in the NV part of Romania and were stored at 4 °C until analysis. The experimental design of our study was developed to characterize the composition of each ingredient in terms of total phenols and flavonoids, phenolic profile, fatty acids, minerals, lipids, proteins and carbohydrates, along with the evaluation of antioxidant capacity. The next step was the elaboration of novel supplements by combining the biological extracts from both materials at different ratios (A1:P1; A1:P2 and A2:P1, *v*/*v*), taking into account the particularities of each one. *Aronia melanocarpa* is a natural source rich in anthocyanins, while bee pollen is rich in proteins. The antioxidant effect of the final product was evaluated in terms of nutritional values and antioxidant capacity, considering the synergic, antagonist or additional effect of the mixture. A schematic presentation of the experimental design is displayed in Figure 2.

### 4.2. Characterization of Raw Materials

#### 4.2.1. Determination of Total Carbohydrate, Lipid and Protein Contents in Aronia and Bee Pollen Samples

Total carbohydrates were determined using the method previously described in the literature by Meryem Bakour et al., 2022 [71]. Briefly, a mixture of 50 μL of bee pollen or Aronia aqueous extracts (1:5, g/v), 150 μL of sulfuric acid (96–98% (*v*/*v*)) and 30 μL of phenol reagent (5%) was heated for 5 min at 90 °C, and then cooled down to room temperature for 5 min. The absorbance of the resulting solution was measured at 492 nm with a microplate reader (Stat Fax 2100, Awareness Technology, Palm City, FL, USA). A calibration curve (R^2^ = 0.995) was obtained using glucose (Merck, Darmstadt, Germany) in the concentration range 10–600 mg/L, while the total carbohydrate content was expressed as gram of glucose equivalents (GlcE)/100 g of the sample.

The total lipid and protein contents in the Aronia and pollen samples were determined based on AOAC 920.85 and AOAC 978.04, respectively [72]. Each measurement was performed in triplicate.

#### 4.2.2. Determination of Mineral Content

In order to determine the major and trace elements concentrations, a closed-vessel Xpert system (Berghof, Eningen, Germany) was used for sample digestion. An amount of 1 g sample was digested using 15 mL 65% HNO_3_ for 2 h, after which it was allowed to reach room temperature. After cooling, a volume of 3 mL H_2_O_2_ 30% was added for a total digestion time of 30 min. Then, the vessels were cooled down, and their volume totaled 50 mL with the addition of ultrapure water. The resulting solutions were analyzed using an inductively coupled plasma optical emission spectrometer Optima 5300DV (ICP-OES, Perkin Elmer, Norwalk, CT, USA) for the determination of major elements.

#### 4.2.3. Determination of Fatty Acids Profile via GS-MS

Each sample was weighed to four decimals, and then placed in a glass tube with 1 mL of n-hexane and 1 mL of 15% BF_3_ in methanol. All the samples were placed in a water bath at 80 °C for 20 min. The samples were neutralized with 1 g of sodium hydrogen sulfate monohydrate, and 1 mL of the upper phase was transferred to a 2 mL vial, and 1 μL of each sample was injected into the Agilent 7890 Gas Chromatograph with the Flame Ionization Detector (Agilent Technologies, Inc. Headquarters, Santa Clara, CA, USA). The separation of the fatty acid methyl esters was carried out with the DB-Wax capillary column (30 m × 0.25 mm i.d. × 0.25 μm film thickness; Agilent, Santa Clara, CA, USA). The injector temperature was set to 250 °C and used in splitless mode; the helium was used as the carrier gas with a flow rate of 1 mL/min. The initial oven temperature was 100 °C and was increased to 180 °C at a rate of 7 °C/min with 5 min of isothermal regime, followed by 180–240 °C at a rate of 10 °C/min with another 10 min of isothermal time. The identification of fatty acid methyl esters (FAME) was achieved by comparing their retention times to the known standards. The results are expressed as % of total fatty acids.

#### 4.2.4. Structural and Morphological Characterization via FTIR and SEM

A ground powder of dried Aronia berries and bee pollen was investigated via FTIR (Fourier Transform Infrared) Spectroscopy in the range 400–4000 cm^−1^ using a Spectrum BXII spectrophotometer (Perkin Elmer) equipped with an MIRacle ATR accessory (ZnSe crystal) at a scanning speed of 32 cm^−1^ and spectral width of 2.0 cm^−1^, with a total of 30 scans. The morphological details were emphasized via scanning electron microscopy (SEM) using a TESCAN VEGA3 device (Libušina, Brno, Czech Republic).

### 4.3. Determination of Bio-Active Compounds in Aronia and Bee POLLEN Samples Using Spectrophotometric Methods

#### 4.3.1. Extraction of Phenols from ARONIA and Bee Pollen

For the extraction of bioactive compounds such as phenols, 2.5 g of dried Aronia was extracted using the Heidolph homogenizer (Heidolph Instruments GmbH & Co., Schwabach, Germany) in 25 mL of MeOH acidified with 0.1% TFA (trifluoroacetic acid). The sample was centrifuged, and the residue was resuspended many times in acidified methanol until all of the anthocyanins were extracted, and the supernatant was used for analysis. To extract phenolic compounds from the pollen sample, 1 g of pollen was extracted with 100 mL EtOH 80%, while shaking on a magnetic stirrer for 48 h, followed by sonication for 10 min, and then centrifugation at 5000 rpm for 20 min. The supernatant was used for analysis.

#### 4.3.2. Total Phenols Content

The Folin–Ciocalteu method was used to determine the total phenolic content (Singleton et al., 1999) [73]. The extract (100 μL) was incubated with 1700 μL of distilled water, 200 μL of Folin–Ciocalteu reagent (freshly prepared) and 1000 μL of 7.5% Na_2_CO_3_ solution for 2 h at room temperature in the dark. Absorbance was measured at 765 nm using a spectrophotometer (Shimadzu 1240 mini UV-Vis, Duisburg, Germany). Different concentrations of gallic acid (0.1–0.5 mg/mL) were used as the standard for reference curve, and the results are expressed in mg gallic acid equivalents (GAE)/g dw [74].

#### 4.3.3. Total Flavonoids Content

An aliquot of each biological extract (1 mL) was transferred to a 10.0 mL volumetric flask containing 4 mL distilled water. the flask was filled with 300 µL of 5% NaNO_2_, and the mixture was allowed to sit for 5 min. Following this, 300 μL AlCl_3_ 10% was added, and after 6 min, 2 mL of 1 M NaOH was added to the mixture, with the flask being filled to exactly 10.0 mL and thoroughly stirred. The absorbance was measured at 510 nm versus a blank. Quercetin was used as the standard for the quantification of total flavonoids, and the results are represented as mg QE (quercetin equivalents)/g dw [74].

#### 4.3.4. Total Monomeric Anthocyanins (Spectrophotometric Method)

The monomeric anthocyanin content of Aronia extracts was measured using the pH differential method [75]. Two dilutions of Aronia extracts were prepared: one in 0.025 M phosphate buffer KCl (pH = 1), and another one in 0.4 M acetate buffer (pH = 4.5).

To calculate the MAP content in Aronia extract, Equation (1) was used:

MAP (mg/L) = (A × MW × DF × 1000)/(ε × 1)(1)

where MW is the molecular weight of cyanidin 3-glucoside (MW = 449.2), DF is the dilution factor, 1000 is the conversion factor for grams to mg, and ε is the molar extinction coefficient (ε = 26,900 L M^−1^ cm^−1^).

### 4.4. Identification and Quantification of Individual Polyphenols via HPLC-DAD-MS-ESI+

In order to extract the phenolic compounds, 1 g of a finely ground powder of dried Aronia berries and bee pollen was added to 10 mL acidified methanol (0.3% HCl) using a homogenizer (Miccra D-9 KT Digitronic, Bergheim, Germany), and then vortexed for 1 min (Heidolph Reax top), followed by 10 min sonication (Elmasonic E 15 H) and centrifugation at 10,000 rot/min at T = 240 °C for 10 min (Eppendorf AG 5804). The procedures were repeated until the complete discoloration of the sample occurred. The supernatant was filtered using a Chromafil Xtra nylon 0.45 µm, and a volume of 20 μL from each sample was injected in the system. HPLC Agilent 1200 equipped with a quaternary pomp, degassing system, autosampler and UV-VIS detector (DAD) coupled with an MS spectrometer (Agilent model 6110, Agilent Technologies, Santa Clara, CA, USA), which was employed for the qualitative and quantitative determination of the bioactive compounds. For separation, a Kinetex XB C18 column (4.6 mm × 150 mm, particle size 5 μm, Phenomenex, Torrance, CA, USA) was used, while the phases were composed of eluent A (water + 0.1% acetic acid) and eluent B (acetonitrile 0.1% acetic acid) using the following gradient schedule (total time 30 min; T = de 25 °C; flow 0.5 mL/min): 0 min and 5% B; 0–2 min and 5% B; 2–18 min and 5–40% B; 18–20 min and 40–90% B; 20–24 min and 90% B; 24–25 min and 90–5% B; 25–30 min and 5% B. The spectral range was 200–600 nm, while the maximum absorbances were recorded at λ = 280, 340 and 520 nm.

The reagents: acetonitrile, HPLC purity, was purchased from Merck (Germany); ultrapure water purified with Direct-Q UV was from Millipore (Bay City, MI, USA); and chlorogenic acid standard (>98% HPLC), gallic acid (>99% HPLC), rutin and cyanidin (>99% HPLC) were purchased from Sigma (St. Louis, MO, USA).

In order to quantify the hydroxybenzoic acids, hydroxycinnamic acids and flavonols, a calibration curve was obtained using gallic acid standard ((R^2^ = 0.9978); LOD = 0.35 μg/mL; LOQ = 1.05 μg/mL), chlorogenic acid standard ((R^2^ = 0.9937); LOD = 0.41 μg/mL; LOQ = 1.64 μg/mL) and rutin standard ((R^2^ = 0.9981); LOD = 0.21 μg/mL; LOQ = 0.84 μg/mL), respectively. Additionally, the quantification of anthocyanins was performed based on calibration curve using cyanidin standard ((R^2^ = 0.9951); LOD = 0.36 μg/mL; LOQ = 1.44 μg/mL). Data acquisition and interpretation were performed using the software Agilent ChemStation, version B.02.01 SR2.

### 4.5. Determination of Antioxidant Capacity of Biological Samples

#### 4.5.1. DPPH (2,2-Diphenyl-1-picryl-hydrazyl-hydrate) Assay

The radical-scavenging capacity of extracts using the stable DPPH radical was determined according to the method in [76], with some modifications. The reduction of DPPH was recorded spectrophotometrically at 517 nm using a Shimadzu mini UV-Vis spectrophotometer. Briefly, a volume of 100 μL of extract was mixed with 2800 μL freshly prepared methanol DPPH solution (80 μM). After exactly 30 min incubation in darkness at room temperature, the absorbance of the sample was measured at 517 nm against a blank of methanol.

The results are expressed in mmolTrolox equivalent (TE)/g dw. The experiment was performed in triplicate, and the results are expressed as average values with a standard deviation (SD).

#### 4.5.2. FRAP (Ferric-Reducing Antioxidant Power) Assay

The antioxidant capacity of the Aronia/pollen extract was evaluated based on the reduction of Fe3+ from tripyridyltriazine Fe(TPTZ)^3+^ complex to the blue-colored complex-Fe(TPTZ)2+ in an acidic medium [77]. The working FRAP solution was freshly prepared by mixing acetate buffer, FeCl_3_•6H_2_O solution and TPTZ solution at the ratio 10:1:1 (*v*/*v*/*v*). The Aronia/pollen extract (100 µL) was allowed to react with 500 µL FRAP working solution and 2 mL distilled water for 1 h in the dark. The final product (ferrous tripyridyltriazine complex) was quantified via VIS absorption (Shimadzu 1240 mini UV-Vis) at 595 nm. The results are expressed in μmol TE/g dw.

#### 4.5.3. TEAC (Trolox Equivalent Antioxidant Capacity) Assay

The TEAC assay is based on the ability of antioxidants to reduce the life of a cation radical (ABTS. +), a green-blue chromophore absorbing at 734 nm [78]. The cation radical was produced by reacting the ABTS (2,2′-azinobis-(3-ethylbenzothiazoline-6-sulfonic acid)) solution (7 mM) with 2.45 mM potassium persulfate, keeping the mixture in the dark at room temperature for 12 h. ABTS stock solution was diluted with ethanol in order to obtain an absorbance of 0.70 ± 0.02 at 734 nm. After the addition of 100 μL Aronia or pollen extract to 2.9 mL of diluted ABTS stock solution, the antioxidant capacity was monitored for exactly 1 min at 734 nm. The results are expressed in μmolTE/g dw.

#### 4.5.4. CUPRAC (Cupric-Reducing Antioxidant Capacity) Assay

This method consists of a combination of 1 mL copper (II) chloride solution (1 × 10^−2^ M), 1 mL neocuproine (2.9-dimethyl-1, 10-phenantroline) alcoholic solution (7.5 × 10^−3^ M), 1 mL ammonium acetate aqueous buffer (pH 7) and 100 µL Aronia/Pollen extract, followed by water to make the final volume 4.1 mL. The absorbance was recorded at 450 nm after 30 min. The results are expressed in μmol TE/g dw [79].

#### 4.5.5. Preparation of Natural Sport Supplements and Determination of Nutritional Values

The starting materials were the extracts of *Aronia melanocarpa* and bee pollen obtained according to the method described in Section 4.3.1. Subsequently, the following combinations were prepared: 1:1, 1:2 and 2:1 (*v*/*v*); the total phenols and flavonoids contents as well as the antioxidant capacity (DPPH, FRAP, TEAC and CUPRAC) of the derived combinations were determined using the methods described in Section 2.5.

The formula established by Nedamani et al., 2015, was used to determine whether a synergistic, additive or antagonistic effect may occur as a result of the combination of the Aronia and pollen extracts in terms of the antioxidant capacity [80].

(2) $$\mathrm{EAC}=\frac{\mathrm{experimental}\;\mathrm{values}\;\mathrm{obtained}\;\mathrm{of}\;\mathrm{antioxidant}\;\mathrm{capacity}}{\mathrm{theoretical}\;\mathrm{values}\;\mathrm{obtained}\;\mathrm{of}\;\mathrm{antioxidant}\;\mathrm{capacity}}$$

EAC—effects of extracts on antioxidant capacity of supplements.

The experimental values represent the real values observed in the case of the combined extracts, while the theoretical ones are calculated as an average of the value observed for each individual extract used in the combinations. A value of EAC > 1 represents a synergistic effect, EAC = 1 represents an additive effect, while EAC < 1 represents an antagonistic effect [80].

The calculation of the energy value was made according to Merrill and Watt [81], taking into account the energy factors for proteins, fats and carbohydrates: 4.00 kcal/g, 8.92 kcal/g and 3.97 kcal/g, respectively [82]. Along with the nutritional values, the antioxidative capacity of each supplement was determined using the methods described in Section 2.4.

### 4.6. Statistical Analysis

All the quantitative measurements were performed in triplicate and are expressed as mean value ± standard deviation (SD). The data were subjected to statistical analysis using one-way ANOVA (Tukey’s multiple comparison test) at the *p* < 0.05 significant level.

## 5. Conclusions

The unique combination of *Aronia melanocarpa* and bee pollen might provide the elements for a personalized sport diet tailored to a particular individual’s requirements. The individualization of supplementation is possible according to each specific sport activity. A better capacity of the organism to cope with intense and repeated training sessions could be sustained via supplementation with the different formulations proposed in our study. The maximum synergism in terms of the antioxidant effect of *Aronia melanocarpa* in combination with bee pollen was demonstrated for the formulation A1:P2. Further clinical studies are necessary to prove the performances of these formulations in vivo and to monitor possible adverse effects.

## Figures and Tables

**Table 1:** Proximate composition of Dried Aronia fruits, bee pollen and different combinations of Aronia and pollen along with the calculated energy values.

Component	Aronia	Bee Pollen	A1:P1	A1:P2	A2:P1
Carbohydrates/100 g	9.80 ± 0.09 ^e^	31.69 ± 0.3 ^a^	20.75 ± 0.2 ^c^	24.39 ± 0.2 ^b^	17.10 ± 0.2 ^d^
Lipids/100 g	13.95 ± 0.9 ^b^	20.44 ± 0.2 ^a,c^	17.20 ± 0.2 ^d,e^	18.28 ± 0.2 ^c,e,f^	16.11 ± 0.2 ^b,d,f^
Proteins/100 g	5.16 ± 0.05 ^e^	23.6 ± 0.2 ^a^	14.38 ± 0.1 ^c^	17.45 ± 0.2 ^b^	11.31 ± 0.1 ^d^
Energy (kcal/100 g)	202.246 ± 2.13 ^d^	402.520 ± 3.89 ^a^	293.448 ± 1.87 ^c,d,b^	329.890 ± 2.31 ^b^	257.005 ± 3.56 ^d^

The results are presented as means ± SD; different letters indicate significant differences within the same line (*p* < 0.05).

**Table 2:** The mineral content (mg/kg) of Aronia and bee pollen samples and different combinations of Aronia and Pollen.

Minerals (mg/Kg/ppm)	Aronia	Bee Pollen	A1:P1	A1:P2	A2:P1
Ca	1270.28	557.32	913.80	794.97	1032.63
K	4054.04	1934.64	2994.34	2641.11	3347.57
Mg	463.88	312.32	388.10	362.84	413.36
Na	200.52	164.28	182.40	176.36	188.44
Fe	28.56	25.68	27.12	26.64	27.60
Zn	2.616	6.824	4.72	5.42	4.02
Mn	17.52	9.44	13.48	12.13	14.83
Si	33.12	26.92	30.02	28.99	31.05
NaCl%	6.76	3.72	5.24	4.73	5.75

**Table 3:** Fatty acids profile (% of total fatty acids).

Fatty Acids	Aronia	Pollen	A1:P1	A1:P2	A2:P1
Caprylic acid (C8:0)	0.91	0.18	1.09	1.27	2.00
Capric acid (C10:0)	0.58	nd	nd	nd	nd
Lauric acid (C12:0)	0.42	0.76	1.19	1.95	1.61
Myristic acid (C14:0)	0.83	0.39	1.22	1.61	2.05
Pentadecenoic acid (C15:1)	0.83	nd	nd	nd	nd
Palmitic acid (C16:0)	8.10	22.12	30.22	52.34	38.32
Palmitoleic acid (C16:1)	15.08	0.15	15.23	15.38	30.31
Heptadecanoic Acid (C17:0)	nd	0.12	nd	nd	nd
Heptadecenoic acid (C17:1)	nd	0.18	nd	nd	nd
Stearic acid (C18:0)	1.37	1.60	2.97	4.57	4.35
Oleic acid (C18:1C+T)	15.61	5.48	21.09	26.56	36.70
Linoleic acid (C18:2C+T) Ɯ-6	43.88	22.76	66.64	89.40	110.52
Linolenic acid (C18:3n3) Ɯ-3	4.28	34.93	39.20	74.13	43.48
Arachidic acid (C20:0)	nd	0.32	nd	nd	nd
9-cis eicosenoic acid (C20:1n9)	nd	0.31	nd	nd	nd
Eicosadienoic acid (C20:2)	nd	0.53	nd	nd	nd
Behenic acid (C22:0)	8.11	0.31	8.42	8.72	16.53
Erucic acid (C22:1n9)	nd	0.43	nd	nd	nd
Docosadienoic acid (C22:2)	nd	10.21	nd	nd	nd
Σ SFA	20.32	25.03	45.36	70.39	65.68
Σ MUFA	30.99	6.55	37.54	44.09	68.53
Σ PUFA	48.16	67.89	116.05	183.94	164.20
Σ SMCFA	1.91	0.18	2.09	2.27	4.00
Σ LCFA	118.41	99.82	218.23	318.06	336.64
PUFAs/SFAs	2.37	2.71	2.56	2.61	2.50
Ɯ-6/n-Ɯ 3 FA	10.26	0.65	1.70	1.21	2.54
UI	52.43	92.61	145.05	237.66	197.48
AI	0.15	0.38	0.25	0.29	0.22
TI	0.20	0.20	0.20	0.20	0.20
h/H	6.82	3.15	4.20	3.77	4.79

Legend: SFA—saturated fatty acid; MUFA—monounsaturated fatty acid, PUFA—polyunsaturated fatty acid, SMCFA—short- and medium-chain fatty acids, LCCFA—Long-Chain Fatty Acid, UI—unsaturated index; AI—index of atherogenicity; TI—index of thrombogenicity; h/H—hypocholesterolemic/hypercholesterolemic (HH) ratio; nd—not determined.

**Table 4:** The total content of bio-active compounds in Aronia and pollen samples determined via spectrophotometric methods.

Sample	Total Phenols Content (mg GAE/g dw)	Total Flavonoids Content (mg QE/g dw)	Total Monomeric Anthocyanin Pigment (MAP) Content (mg/L)
Aronia	17.56 ± 3.49 ^a^	87.68 ± 9.86 ^a^	192.371 ± 22.67
Pollen	22.62 ± 0.88 ^a^	85.75 ± 15.30 ^a^	-
A1:P1 (*v*/*v*)	24.80 ± 4.99 ^a^	87.17 ± 15.55 ^a^	-
A1:P2 (*v*/*v*)	24.08 ± 6.52 ^a^	92.99 ± 14.26 ^a^	-
A2:P1 (*v*/*v*)	22.27 ± 5.76 ^a^	104.17 ± 19.81 ^a^	-

Results are represented as means ± SD; the same letters indicate no significant differences within the column (*p* < 0.05).

**Table 5:** HPLC-DAD-MS (ESI+) tentative phenolic compounds identified and quantified (mg/g dw) in the Aronia sample.

Peak No.	R_t_ (min)	UV λ_max_ (nm)	[M+H]^+^ (*m*/*z*)	Compound	Subclass	Sample: Aronia * (mg/g)
1	2.99	280	139	2-Hydroxybenzoic acid	Hydroxybenzoic acid	1.531 ± 0.09
2	4.03	280	155	Dihydroxybenzoic acid	Hydroxybenzoic acid	0.675 ± 0.03
3	10.21	280	155	Protocatechuic acid	Hydroxybenzoic acid	1.094 ± 0.07
4	10.99	520,280	449	Cyanidin-glucoside	Anthocyanin	0.257 ± 0.03
5	11.70	519,279	419,287	Cyanidin-arabinoside	Anthocyanin	0.239 ± 0.01
6	12.43	519,279	419,287	Cyanidin-xyloside	Anthocyanin	0.199 ± 0.01
7	13.04	332	355	5-Caffeoylquinic acid (Chlorogenic acid)	Hydroxycinnamic acid	3.421 ± 0.2
8	13.58	330	181,163	Caffeic acid	Hydroxycinnamic acid	4.971 ± 0.2
9	14.89	333	369	3-Feruloylquinic acid	Hydroxycinnamic acid	3.130 ± 0.1
10	15.55	333	369	5-Feruloylquinic acid	Hydroxycinnamic acid	9.049 ± 0.08
11	15.83	355,250	611,303	Quercetin-rutinoside (Rutin)	Flavonol	0.906 ± 0.07
12	16.39	354,250	465,303	Quercetin-glucoside	Flavonol	2.115 ± 0.01
13	21.81	356,251	303	Quercetin	Flavonol	0.431 ± 0.05
				Total phenols		28.017

* The values are presented as mean ± SD, (*n* = 3). Rt—retention time.

**Table 6:** Identification and quantification of phenolic compounds in bee pollen sample (HPLC-DAD-MS (ESI^+^).

Peak No.	R_t_ (min)	UV λ_max_ (nm)	[M+H]^+^ (*m*/*z*)	Compound	Subclass	Sample: Pollen * (mg/g)
1	3.00	280	139	2-Hydroxybenzoic acid	Hydroxybenzoic acid	2.753 ± 0.19
2	4.05	280	155	Dihydroxybenzoic acid	Hydroxybenzoic acid	0.843 ± 0.01
3	12.96	332	355	5-Caffeoylquinic acid (Chlorogenic acid)	Hydroxycinnamic acid	0.304 ± 0.02
4	14.58	354, 250	627,303	Quercetin-diglucoside	Flavonol	4.416 ± 0.65
5	15.07	350, 255	641,317	Isorhamnetin-diglucoside	Flavonol	1.509 ± 0.9
6	15.41	350, 250	757,611, 449,287	Kaempferol-glucoside-glucoside-rhamnoside	Flavonol	4.272 ± 0.11
7	15.81	355, 250	611,303	Quercetin-rutinoside (Rutin)	Flavonol	4.654 ± 0.13
8	16.09	350, 250	595,287	Kaempferol-rutinoside	Flavonol	3.548 ± 0.14
9	16.54	330	339	*p*-Coumaroylquinic acid	Hydroxycinnamic acid	0.629 ± 0.02
10	17.25	350, 255	479,317	Isorhamnetin-glucoside	Flavonol	4.125 ± 0.21
11	17.54	350, 250	449,287	Kaempferol-glucoside	Flavonol	1.051 ± 0.099
12	18.54	350, 250	565,287	Kaempferol-rhamnoside-arabinoside	Flavonol	3.333 ± 0.13
13	19.23	322	674	Triferuloyl spermidine	Hydroxycinnamic acid amide derivative	4.422 ± 0.28
14	20.28	322	644	Diferuloyl-coumaroyl spermidine	Hydroxycinnamic acid amide derivative	11.926 ± 0.99
15	21.15	320	584	Tricoumaroyl spermidine	Hydroxycinnamic acid amide derivative	3.409 ± 0.26
16	22.31	321	614	Feruloyl-dicoumaroyl spermidine	Hydroxycinnamic acid amide derivative	5.002 ± 0.13
				Total phenols		56.197

* The values are presented as mean ± SD, (*n* = 3). Rt—retention time.

**Table 7:** Antioxidant capacity of Dried Aronia fruits, bee pollen and different combinations.

	Aronia	Pollen	A1:P1 (*v*/*v*)	A1:P2 (*v*/*v*)	A2:P1 (*v*/*v*)
DPPH (mmolTE/g)	350.95 ± 0.49 ^d^	413.85 ± 0.51 ^a^	380.61 ± 8.32 ^b,c^	391.27 ± 0.52 ^b,c^	373.62 ± 2.60 ^b^
EAC	-	-	0.995	1.023	0.977
			Additional	Synergic	Additional
FRAP (µmolTE/g)	66.32 ± 8.65 ^a^	36.56 ± 11.26 ^b^	71.57 ± 0.26 ^a,b,c^	61.27 ± 0.78 ^a,b^	76.72 ± 0.78 ^a,c^
EAC	-	-	1.39	1.19	1.49
			Synergic	Synergic	Synergic
TEAC (µmol TE/g)	63.24 ± 16.06 ^d^	86.06 ± 1.53 ^c^	85.56 ± 7.37 ^a,b,c,d^	89.40 ± 1.16 ^a^	65.00 ± 8.53 ^b^
EAC	-	-	1.146	1.198	0.871
			Synergic	Synergic	Antagonist
CUPRAC (µmol TE/g)	189.66 ± 0.87 ^c^	155.18 ± 23.63 ^c^	209.36 ± 26.07 ^a^	176.71 ± 13.26 ^c^	160.35 ± 9.24 ^b,c^
EAC	-	-	1.214	1.025	0.930
			Synergic	Synergic	Antagonist

Legend: AC—effects of extracts on antioxidant capacity of supplements. Results are presented as means ± SD; different letters indicate significant differences within the same line (*p* < 0.05).

## Data Availability

Not applicable.

## Supplementary Materials

The following supporting information can be downloaded at: https://www.mdpi.com/article/10.3390/molecules28196944/s1, Figure S1. HPLC chromatograms of Aronia sample recorded at different wavelengths: (a) 280 nm, (b) 340 nm and (c) 520 nm; Figure S2. HPLC chromatograms of pollen sample recorded at different wavelength. (a) 280 nm, (b) 340 nm.

## References

[1] Maughan R.J., Burke L.M., Dvorak J., Larson-Meyer D.E., Peeling P., Phillips S.M., Rawson E.S., Walsh N.P., Garthe I., Geyer H. (2018). IOC Consensus Statement: Dietary Supplements and the High-Performance Athlete. Int. J. Sport. Nutr. Exerc. Metab..

[2] Lindschinger M., Tatzber F., Schimetta W., Schmid I., Lindschinger B., Cvirn G., Fuchs N., Markolin G., Lamont E., Wonisch W. (2020). Bioavailability of natural versus synthetic B vitamins and their effects on metabolic processes. MMW Fortschr. Med..

[3] Bachmann H., Offord-Cavin E., Phothirath P., Horcajada M.-N., Romeis P., Mathis G.A. (2013). 1,25-Dihydroxyvitamin D3-Glycoside of Herbal Origin Exhibits Delayed Release Pharmacokinetics When Compared to Its Synthetic Counterpart. J. Steroid Biochem. Mol. Biol..

[4] Berger R.G., Lunkenbein S., Ströhle A., Hahn A. (2012). Antioxidants in Food: Mere Myth or Magic Medicine?. Crit. Rev. Food Sci. Nutr..

[5] Denisow B., Denisow-Pietrzyk M. (2016). Biological and Therapeutic Properties of Bee Pollen: A Review. J. Sci. Food Agric..

[6] Dickson J.H., Oeggl K., Holden T.G., Handley L.L., O’Connell T.C., Preston T. (2000). The Omnivorous Tyrolean Iceman: Colon Contents (Meat, Cereals, Pollen, Moss and Whipworm) and Stable Isotope Analyses. Philos. Trans. R. Soc. Lond. B Biol. Sci..

[7] Martín-Muñoz M.F., Bartolome B., Caminoa M., Bobolea I., Garcia Ara M.C., Quirce S. (2010). Bee Pollen: A Dangerous Food for Allergic Children. Identification of Responsible Allergens. Allergol. Immunopathol..

[8] Algethami J.S., El-Wahed A.A.A., Elashal M.H., Ahmed H.R., Elshafiey E.H., Omar E.M., Naggar Y.A., Algethami A.F., Shou Q., Alsharif S.M. (2022). Bee Pollen: Clinical Trials and Patent Applications. Nutrients.

[9] Ali A.M., Kunugi H. (2020). Apitherapy for Age-Related Skeletal Muscle Dysfunction (Sarcopenia): A Review on the Effects of Royal Jelly, Propolis, and Bee Pollen. Foods.

[10] Taha E.-K.A., Al-Kahtani S., Taha R. (2019). Protein Content and Amino Acids Composition of Bee-Pollens from Major Floral Sources in Al-Ahsa, Eastern Saudi Arabia. Saudi J. Biol. Sci..

[11] Jeannerod L., Carlier A., Schatz B., Daise C., Richel A., Agnan Y., Baude M., Jacquemart A.-L. (2022). Some Bee-Pollinated Plants Provide Nutritionally Incomplete Pollen Amino Acid Resources to Their Pollinators. PLoS ONE.

[12] Oroian M., Dranca F., Ursachi F. (2022). Characterization of Romanian Bee Pollen—An Important Nutritional Source. Foods.

[13] Maughan R.J., Evans S.P. (1982). Effects of Pollen Extract upon Adolescent Swimmers. Br. J. Sports Med..

[14] Steben R.E., Boudreaux P. (1978). The Effects of Pollen and Protein Extracts on Selected Blood Factors and Performance of Athletes. J. Sports Med. Phys. Fit..

[15] Nazarian A., Azarbayjani M.A., Atashak S., Peeri M. (2022). Effects of Resistance Training, Palm Pollen Grain Extracts, and Testosterone Injection on Luteinizing Hormone Receptors, Claudin-1, Cingulin, and Zonula Occludens in the Prostate Tissues of Adult Male Rats. Andrologia.

[16] Ketkar S., Rathore A., Kandhare A., Lohidasan S., Bodhankar S., Paradkar A., Mahadik K. (2015). Alleviating Exercise-Induced Muscular Stress Using Neat and Processed Bee Pollen: Oxidative Markers, Mitochondrial Enzymes, and Myostatin Expression in Rats. Integr. Med. Res..

[17] Salles J., Cardinault N., Patrac V., Berry A., Giraudet C., Collin M.-L., Chanet A., Tagliaferri C., Denis P., Pouyet C. (2014). Bee Pollen Improves Muscle Protein and Energy Metabolism in Malnourished Old Rats through Interfering with the Mtor Signaling Pathway and Mitochondrial Activity. Nutrients.

[18] Elghouizi A., Al-Waili N., Elmenyiy N., Elfetri S., Aboulghazi A., Al-Waili A., Lyoussi B. (2022). Protective Effect of Bee Pollen in Acute Kidney Injury, Proteinuria, and Crystalluria Induced by Ethylene Glycol Ingestion in Rats. Sci. Rep..

[19] Turner K.K., Nielsen B.D., O’Connor C.I., Burton J.L. (2006). Bee Pollen Product Supplementation to Horses in Training Seems to Improve Feed Intake: A Pilot Study. J. Anim. Physiol. Anim. Nutr..

[20] Jurendić T., Ščetar M. (2021). Aronia Melanocarpa Products and By-Products for Health and Nutrition: A Review. Antioxidants.

[21] Ren Y., Frank T., Meyer G., Lei J., Grebenc J.R., Slaughter R., Gao Y.G., Kinghorn A.D. (2022). Potential Benefits of Black Chokeberry (*Aronia melanocarpa*) Fruits and Their Constituents in Improving Human Health. Molecules.

[22] Powers S.K., Deminice R., Ozdemir M., Yoshihara T., Bomkamp M.P., Hyatt H. (2020). Exercise-Induced Oxidative Stress: Friend or Foe?. J. Sport. Health Sci..

[23] Zare R., Kimble R., Ali Redha A., Cerullo G., Clifford T. (2023). How Can Chokeberry (*Aronia*) (Poly)Phenol-Rich Supplementation Help Athletes? A Systematic Review of Human Clinical Trials. Food Funct..

[24] Stankiewicz B., Cieślicka M., Mieszkowski J., Kochanowicz A., Niespodziński B., Szwarc A., Waldziński T., Reczkowicz J., Piskorska E., Petr M. (2023). Effect of Supplementation with Black Chokeberry (*Aronia melanocarpa*) Extract on Inflammatory Status and Selected Markers of Iron Metabolism in Young Football Players: A Randomized Double-Blind Trial. Nutrients.

[25] Cikiriz N., Milosavljevic I., Jakovljevic B., Bolevich S., Jeremic J., Nikolic Turnic T., Mitrovic M., Srejovic I., Bolevich S., Jakovljevic V. (2021). The Influences of Chokeberry Extract Supplementation on Redox Status and Body Composition in Handball Players during Competition Phase. Can. J. Physiol. Pharmacol..

[26] Pilaczynska-Szczesniak L., Skarpanska-Steinborn A., Deskur E., Basta P., Horoszkiewicz-Hassan M. (2005). The Influence of Chokeberry Juice Supplementation on the Reduction of Oxidative Stress Resulting from an Incremental Rowing Ergometer Exercise. Int. J. Sport. Nutr. Exerc. Metab..

[27] Tirla A., Islam F., Islam M.R., Ioana Vicas S., Cavalu S. (2022). New Insight and Future Perspectives on Nutraceuticals for Improving Sports Performance of Combat Players: Focus on Natural Supplements, Importance and Advantages over Synthetic Ones. Appl. Sci..

[28] Timmons J.S., Weiss W.P., Palmquist D.L., Harper W.J. (2001). Relationships Among Dietary Roasted Soybeans, Milk Components, and Spontaneous Oxidized Flavor of Milk1. J. Dairy. Sci..

[29] Ulbricht T.L.V., Southgate D.A.T. (1991). Coronary Heart Disease: Seven Dietary Factors. Lancet.

[30] Ćujić N., Trifković K., Bugarski B., Ibrić S., Pljevljakušić D., Šavikin K. (2016). Chokeberry (*Aronia melanocarpa* L.) Extract Loaded in Alginate and Alginate/Inulin System. Ind. Crops Prod..

[31] Prđun S., Svečnjak L., Valentić M., Marijanović Z., Jerković I. (2021). Characterization of Bee Pollen: Physico-Chemical Properties, Headspace Composition and FTIR Spectral Profiles. Foods.

[32] Lahlali R., Jiang Y., Kumar S., Karunakaran C., Liu X., Borondics F., Hallin E., Bueckert R. (2014). ATR–FTIR Spectroscopy Reveals Involvement of Lipids and Proteins of Intact Pea Pollen Grains to Heat Stress Tolerance. Front. Plant Sci..

[33] Memete A.R., Miere (Groza) F., Laslo V., Purcarea C., Vicas L., Ganea M., Antonescu A., Vicas S.I. (2023). An In Vitro Study of the Healing Potential of Black Mulberry (*Morus nigra* L.) Extract in a Liposomal Formulation. Appl. Sci..

[34] Aylanc V., Tomás A., Russo-Almeida P., Falcão S.I., Vilas-Boas M. (2021). Assessment of Bioactive Compounds under Simulated Gastrointestinal Digestion of Bee Pollen and Bee Bread: Bioaccessibility and Antioxidant Activity. Antioxidants.

[35] Edreva A.M., Velikova V.B., Tsonev T.D. (2007). Phenylamides in Plants. Russ. J. Plant Physiol..

[36] Stokes T., Hector A.J., Morton R.W., McGlory C., Phillips S.M. (2018). Recent Perspectives Regarding the Role of Dietary Protein for the Promotion of Muscle Hypertrophy with Resistance Exercise Training. Nutrients.

[37] Poulios A., Georgakouli K., Draganidis D., Deli C.K., Tsimeas P.D., Chatzinikolaou A., Papanikolaou K., Batrakoulis A., Mohr M., Jamurtas A.Z. (2019). Protein-Based Supplementation to Enhance Recovery in Team Sports: What Is the Evidence?. J. Sports Sci. Med..

[38] Sierra-Galicia M.I., Rodríguez-de Lara R., Orzuna-Orzuna J.F., Lara-Bueno A., Ramírez-Valverde R., Fallas-López M. (2023). Effects of Supplementation with Bee Pollen and Propolis on Growth Performance and Serum Metabolites of Rabbits: A Meta-Analysis. Animals.

[39] Wang J., Li S., Wang Q., Xin B., Wang H. (2007). Trophic Effect of Bee Pollen on Small Intestine in Broiler Chickens. J. Med. Food.

[40] Abdelnour S.A., Abd El-Hack M.E., Alagawany M., Farag M.R., Elnesr S.S. (2019). Beneficial Impacts of Bee Pollen in Animal Production, Reproduction and Health. J. Anim. Physiol. Anim. Nutr..

[41] HaščíK P., Pavelková A., Bobko M., Trembecká L., Elimam I.O.E., Capcarová M. (2017). The Effect of Bee Pollen in Chicken Diet. World’s Poult. Sci. J..

[42] Williams C., Rollo I. (2015). Carbohydrate Nutrition and Team Sport Performance. Sports Med..

[43] Gonzalez J.T., Wallis G.A. (2021). Carb-Conscious: The Role of Carbohydrate Intake in Recovery from Exercise. Curr. Opin. Clin. Nutr. Metab. Care.

[44] Newell M., Wallis G., Hunter A., Tipton K., Galloway S. (2018). Metabolic Responses to Carbohydrate Ingestion during Exercise: Associations between Carbohydrate Dose and Endurance Performance. Nutrients.

[45] Rowlands D.S., Houltham S., Musa-Veloso K., Brown F., Paulionis L., Bailey D. (2015). Fructose-Glucose Composite Carbohydrates and Endurance Performance: Critical Review and Future Perspectives. Sports Med..

[46] Fuchs C.J., Gonzalez J.T., Loon L.J.C. (2019). Fructose Co-ingestion to Increase Carbohydrate Availability in Athletes. J. Physiol..

[47] Starowicz M., Hanus P., Lamparski G., Sawicki T. (2021). Characterizing the Volatile and Sensory Profiles, and Sugar Content of Beeswax, Beebread, Bee Pollen, and Honey. Molecules.

[48] Thielecke F., Blannin A. (2020). Omega-3 Fatty Acids for Sport Performance—Are They Equally Beneficial for Athletes and Amateurs? A Narrative Review. Nutrients.

[49] Zevenbergen H., de Bree A., Zeelenberg M., Laitinen K., van Duijn G., Flöter E. (2009). Foods with a High Fat Quality Are Essential for Healthy Diets. Ann. Nutr. Metab..

[50] Thomas D.T., Erdman K.A., Burke L.M. (2016). Position of the Academy of Nutrition and Dietetics, Dietitians of Canada, and the American College of Sports Medicine: Nutrition and Athletic Performance. J. Acad. Nutr. Diet..

[51] Burke L.M., Ross M.L., Garvican-Lewis L.A., Welvaert M., Heikura I.A., Forbes S.G., Mirtschin J.G., Cato L.E., Strobel N., Sharma A.P. (2017). Low Carbohydrate, High Fat Diet Impairs Exercise Economy and Negates the Performance Benefit from Intensified Training in Elite Race Walkers. J. Physiol..

[52] Goldenshluger A., Constantini K., Goldstein N., Shelef I., Schwarzfuchs D., Zelicha H., Yaskolka Meir A., Tsaban G., Chassidim Y., Gepner Y. (2021). Effect of Dietary Strategies on Respiratory Quotient and Its Association with Clinical Parameters and Organ Fat Loss: A Randomized Controlled Trial. Nutrients.

[53] Philpott J.D., Witard O.C., Galloway S.D.R. (2019). Applications of Omega-3 Polyunsaturated Fatty Acid Supplementation for Sport Performance. Res. Sports Med..

[54] Simopoulos A.P. (2002). The Importance of the Ratio of Omega-6/Omega-3 Essential Fatty Acids. Biomed. Pharmacother..

[55] Chen J., Liu H. (2020). Nutritional Indices for Assessing Fatty Acids: A Mini-Review. Int. J. Mol. Sci..

[56] Moussavi Javardi M.S., Madani Z., Movahedi A., Karandish M., Abbasi B. (2020). The Correlation between Dietary Fat Quality Indices and Lipid Profile with Atherogenic Index of Plasma in Obese and Non-Obese Volunteers: A Cross-Sectional Descriptive-Analytic Case-Control Study. Lipids Health Dis..

[57] Adams M., Fell J., Williams A. (2009). Exercise Causing Thrombosis. Physician Sportsmed..

[58] Tirla A., Vesa C.M., Cavalu S. (2021). Severe Cardiac and Metabolic Pathology Induced by Steroid Abuse in a Young Individual. Diagnostics.

[59] Grozenski A., Kiel J. (2020). Basic Nutrition for Sports Participation, Part 2: Vitamins and Minerals. Curr. Sports Med. Rep..

[60] Heffernan S., Horner K., De Vito G., Conway G. (2019). The Role of Mineral and Trace Element Supplementation in Exercise and Athletic Performance: A Systematic Review. Nutrients.

[61] Houston M.C., Harper K.J. (2008). Potassium, Magnesium, and Calcium: Their Role in Both the Cause and Treatment of Hypertension. J. Clin. Hypertens..

[62] Belenguer-Varea Á., Tarazona-Santabalbina F.J., Avellana-Zaragoza J.A., Martínez-Reig M., Mas-Bargues C., Inglés M. (2020). Oxidative Stress and Exceptional Human Longevity: Systematic Review. Free Radic. Biol. Med..

[63] Hajam Y.A., Rani R., Ganie S.Y., Sheikh T.A., Javaid D., Qadri S.S., Pramodh S., Alsulimani A., Alkhanani M.F., Harakeh S. (2022). Oxidative Stress in Human Pathology and Aging: Molecular Mechanisms and Perspectives. Cells.

[64] Xu Y., Liang M., Ugbolue U.C., Fekete G., Gu Y. (2022). Effect of Physical Exercise Under Different Intensity and Antioxidative Supplementation for Plasma Superoxide Dismutase in Healthy Adults: Systematic Review and Network Meta-Analysis. Front. Physiol..

[65] Cobley J.N., Close G.L., Bailey D.M., Davison G.W. (2017). Exercise Redox Biochemistry: Conceptual, Methodological and Technical Recommendations. Redox Biol..

[66] Zhou Z., Chen C., Teo E.-C., Zhang Y., Huang J., Xu Y., Gu Y. (2022). Intracellular Oxidative Stress Induced by Physical Exercise in Adults: Systematic Review and Meta-Analysis. Antioxidants.

[67] Margaritelis N.V., Paschalis V., Theodorou A.A., Kyparos A., Nikolaidis M.G. (2020). Redox Basis of Exercise Physiology. Redox Biol..

[68] Canals-Garzón C., Guisado-Barrilao R., Martínez-García D., Chirosa-Ríos I.J., Jerez-Mayorga D., Guisado-Requena I.M. (2022). Effect of Antioxidant Supplementation on Markers of Oxidative Stress and Muscle Damage after Strength Exercise: A Systematic Review. Int. J. Environ. Res. Public Health.

[69] Gulcin İ. (2020). Antioxidants and Antioxidant Methods: An Updated Overview. Arch. Toxicol..

[70] Christodoulou M.C., Orellana Palacios J.C., Hesami G., Jafarzadeh S., Lorenzo J.M., Domínguez R., Moreno A., Hadidi M. (2022). Spectrophotometric Methods for Measurement of Antioxidant Activity in Food and Pharmaceuticals. Antioxidants.

[71] Bakour M., Laaroussi H., Ferreira-Santos P., Genisheva Z., Ousaaid D., Teixeira J.A., Lyoussi B. (2022). Exploring the Palynological, Chemical, and Bioactive Properties of Non-Studied Bee Pollen and Honey from Morocco. Molecules.

[72] Horowitz W., Latimer G. (2006). Official Methods of Analysis of AOAC International.

[73] Singleton V.L., Orthofer R., Lamuela-Raventós R.M. (1999). Analysis of Total Phenols and other Oxidation Substrates and Antioxidants by Means of Folin-Ciocalteu Reagent. Methods in Enzymology.

[74] Memete A.R., Sărac I., Teusdea A.C., Budău R., Bei M., Vicas S.I. (2023). Bioactive Compounds and Antioxidant Capacity of Several Blackberry (*Rubus* Spp.) Fruits Cultivars Grown in Romania. Horticulturae.

[75] Giusti M.M., Wrolstad R.E. (2001). Characterization and Measurement of Anthocyanins by UV-Visible Spectroscopy. Curr. Protoc. Food Anal. Chem..

[76] Brand-Williams W., Cuvelier M.E., Berset C. (1995). Use of a Free Radical Method to Evaluate Antioxidant Activity. LWT Food Sci. Technol..

[77] Benzie I.F.F., Strain J.J. (1996). The Ferric Reducing Ability of Plasma (FRAP) as a Measure of “Antioxidant Power”: The FRAP Assay. Anal. Biochem..

[78] Vicaş S.I., Bandici L., Teuşdea A.C., Turcin V., Popa D., Bandici G.E. (2017). The Bioactive Compounds, Antioxidant Capacity, and Color Intensity in Must and Wines Derived from Grapes Processed by Pulsed Electric Field. CyTA J. Food.

[79] Apak R., Özyürek M., Güçlü K., Çapanoğlu E. (2016). Antioxidant Activity/Capacity Measurement. 1. Classification, Physicochemical Principles, Mechanisms, and Electron Transfer (ET)-Based Assays. J. Agric. Food Chem..

[80] Ranjbar Nedamani E., Sadeghi Mahoonak A., Ghorbani M., Kashaninejad M. (2015). Evaluation of Antioxidant Interactions in Combined Extracts of Green Tea (*Camellia sinensis*), Rosemary (*Rosmarinus officinalis*) and Oak Fruit (*Quercus branti*). J. Food Sci. Technol..

[81] Merrill A.L., Watt B.K. (1955). Energy Value of Foods: Basis and Derivation.

[82] Capuano E., Oliviero T., Fogliano V., Pellegrini N. (2018). Role of the Food Matrix and Digestion on Calculation of the Actual Energy Content of Food. Nutr. Rev..

