# Supplementary material for: Designing New Sport Supplements Based on Aronia melanocarpa and Bee Pollen to Enhance Antioxidant Capacity and Nutritional Value
Source: Molecules. 2023 Oct 5;28(19):6944. doi: 10.3390/molecules28196944 (PMC10574696; doi:10.3390/molecules28196944)
Supplement: Supplementary file 1 [file molecules-28-06944-s001.zip › molecules-2589713-supplementary.pdf]

Supplementary material

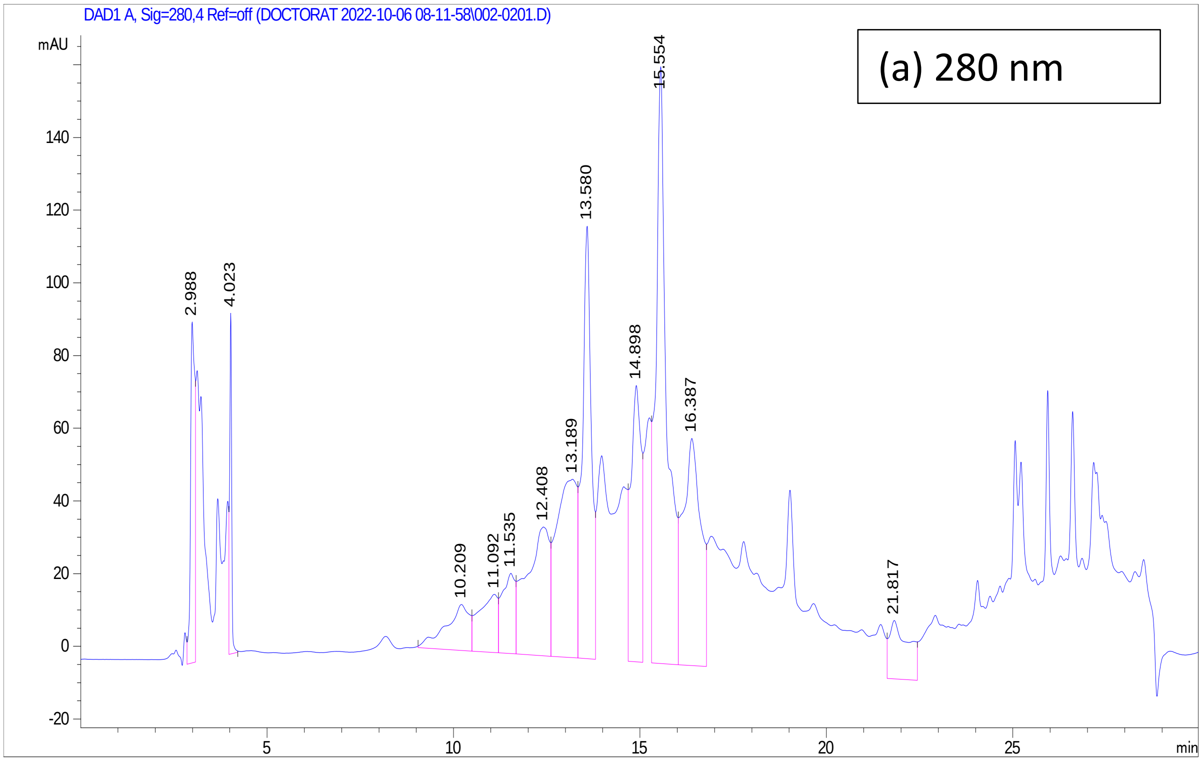

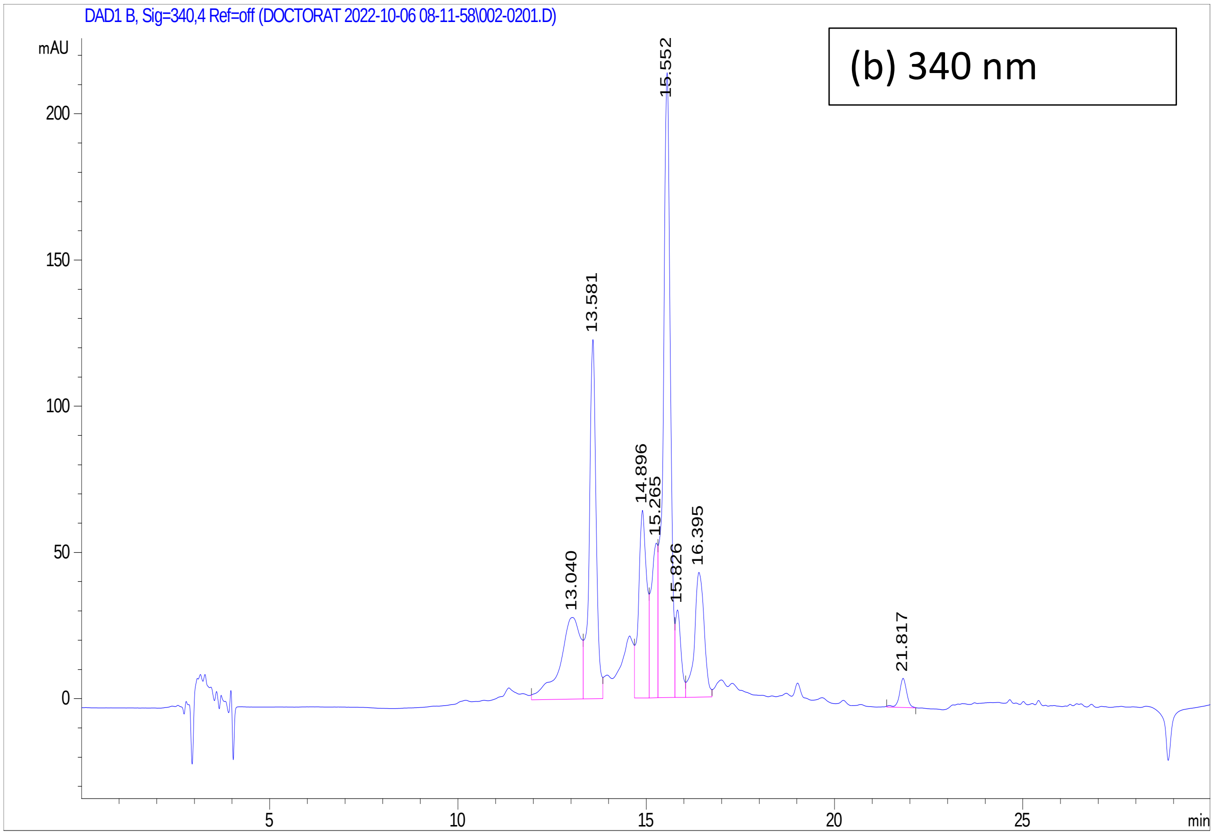

Supplementary material

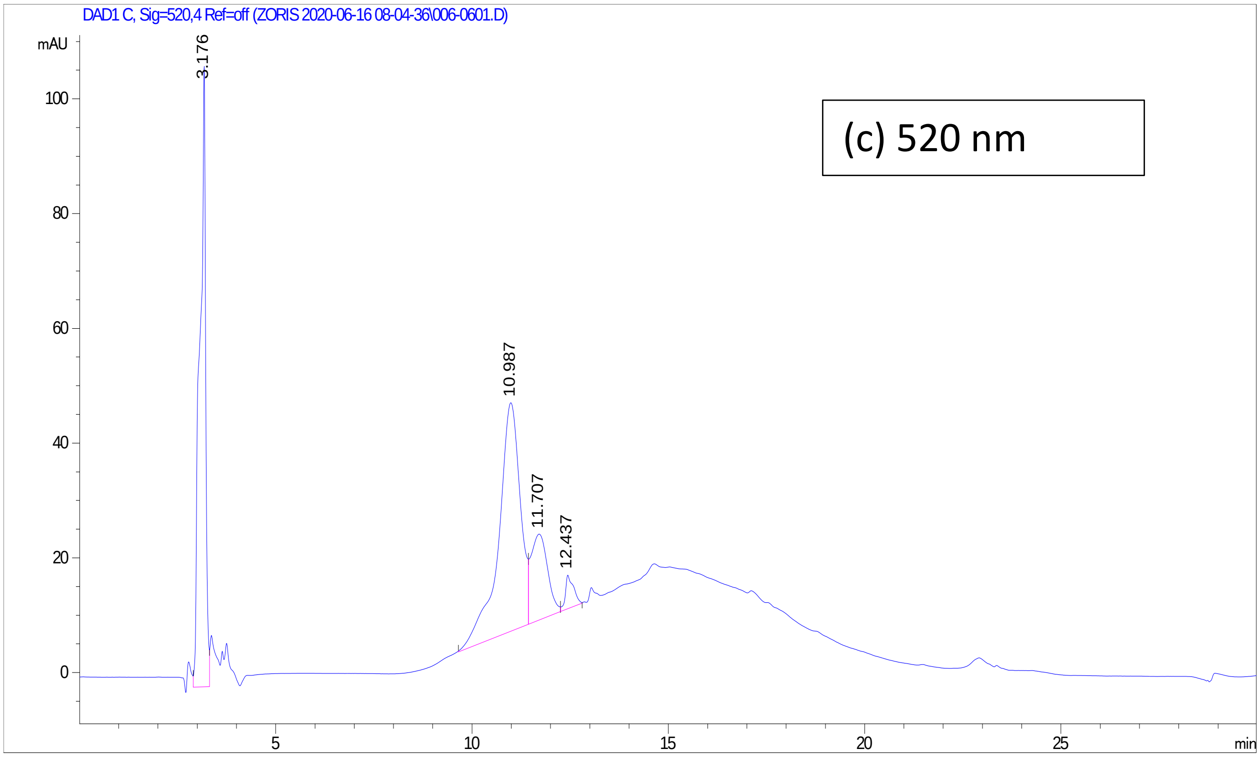

Figure S1. HPLC chromatograms of Aronia sample recorded at different wavelength. (**a**) 280 nm; (**b**) 340 nm and (**c**) 520 nm.

Supplementary material

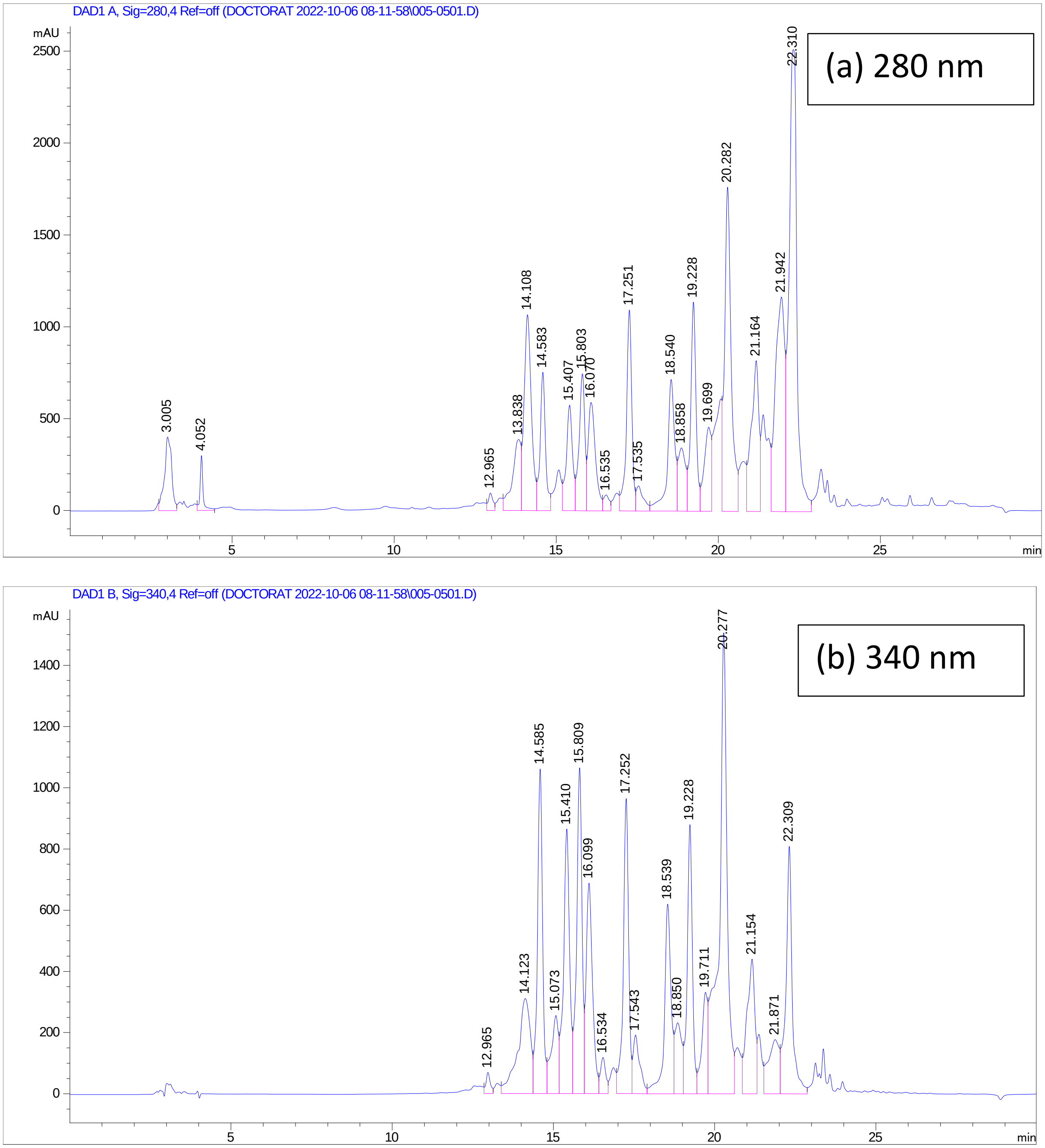

Figure S2. HPLC chromatograms of pollen sample recorded at different wavelength: (**a**) 280 nm and (**b**) 340 nm.
